# C-Reactive Protein and N-Terminal Pro-brain Natriuretic Peptide Levels Correlate With Impaired Cardiorespiratory Fitness in Patients With Heart Failure Across a Wide Range of Ejection Fraction

**DOI:** 10.3389/fcvm.2018.00178

**Published:** 2018-12-21

**Authors:** Jessie van Wezenbeek, Justin M. Canada, Krishna Ravindra, Salvatore Carbone, Cory R. Trankle, Dinesh Kadariya, Leo F. Buckley, Marco Del Buono, Hayley Billingsley, Michele Viscusi, George F. Wohlford, Ross Arena, Benjamin Van Tassell, Antonio Abbate

**Affiliations:** ^1^VCU Pauley Heart Center, Virginia Commonwealth University, Richmond, VA, United States; ^2^Department of Pharmacotherapy and Outcome Sciences, Virginia Commonwealth University, Richmond, VA, United States; ^3^Department of Physical Therapy, College of Applied Health Sciences, University of Illinois, Chicago, IL, United States

**Keywords:** heart failure, biomarker, systemic inflammation, myocardial strain, cardiorespiratory fitness, cardiopulmonary exercise testing

## Abstract

**Background:** Impaired cardiorespiratory fitness (CRF) is a hallmark of heart failure (HF). Serum levels of C-reactive protein (CRP), a systemic inflammatory marker, and of N-terminal pro-brain natriuretic peptide (NT-proBNP), a biomarker of myocardial strain, independently predict adverse outcomes in HF patients. Whether CRP and/or NT-proBNP also predict the degree of CRF impairment in HF patients across a wide range of ejection fraction is not yet established.

**Methods:** Using retrospective analysis, 200 patients with symptomatic HF who completed one or more treadmill cardiopulmonary exercise tests (CPX) using a symptom-limited ramp protocol and had paired measurements of serum high-sensitivity CRP and NT-proBNP on the same day were evaluated. Univariate and multivariate correlations were evaluated with linear regression after logarithmic transformation of CRP (log_10_) and NT-proBNP (log_N_).

**Results:** Mean age of patients was 57 ± 10 years and 55% were male. Median CRP levels were 3.7 [1.5–9.0] mg/L, and NT-proBNP levels were 377 [106–1,464] pg/ml, respectively. Mean peak oxygen consumption (peak VO_2_) was 16 ± 4 mlO_2_•kg^−1^•min^−1^. CRP levels significantly correlated with peakVO_2_ in all patients (*R* = −0.350, *p* < 0.001) and also separately in the subgroup of patients with reduced left ventricular ejection fraction (LVEF) (HFrEF, *N* = 109) (*R* = −0.282, *p* < 0.001) and in those with preserved EF (HFpEF, *N* = 57) (*R* = −0.459, *p* < 0.001). NT-proBNP levels also significantly correlated with peak VO_2_ in all patients (*R* = −0.330, *p* < 0.001) and separately in patients with HFrEF (*R* = −0.342, *p* < 0.001) and HFpEF (*R* = −0.275, *p* = 0.032). CRP and NT-proBNP did not correlate with each other (*R* = 0.05, *p* = 0.426), but independently predicted peak VO_2_ (*R* = 0.421, *p* < 0.001 and *p* < 0.001, respectively).

**Conclusions:** Biomarkers of inflammation and myocardial strain independently predict peak VO_2_ in HF patients. Anti-inflammatory therapies and therapies alleviating myocardial strain may independently improve CRF in HF patients across a large spectrum of LVEF.

## Introduction

Heart failure (HF) is a syndrome that presents clinically with dyspnea, fatigue, and/or edema caused by structural or functional cardiac defects that lead to reduced cardiac output and/or increased cardiac pressure at rest or during stress. Impaired cardiorespiratory fitness (CRF) is a hallmark of heart failure (HF) (1). CRF is defined as the ability of the circulatory, respiratory, and muscular systems to supply oxygen during sustained physical activity (2). CRF is expressed in metabolic equivalents (METs) and measured by peak oxygen uptake (peak VO_2_) using exercise tests (3). CRF is both an objective measure of habitual physical activity, as well as a prognostic indicator in HF.

C-reactive protein (CRP), a marker for systemic inflammation, is produced by the hepatocytes upon inflammation, infection, or tissue injury (4). Patients with HF show signs of chronic systemic inflammation, as shown by elevated serum levels of CRP (5). Increased levels of CRP are associated with an increased risk for CVD events and for mortality (6, 7). Higher CRP levels are also associated with worse cardiopulmonary exercise performance in patients with ischemic heart disease and systolic HF (8, 9).

Natriuretic peptides are peptide hormones that function as counter-regulatory mechanisms for the Renin-Angiotensin-Aldosterone-system (RAAS), and therefore cause a decrease in arterial pressure, central venous pressure, pulmonary capillary wedge pressure, cardiac output, and total blood volume through natriuresis and diuresis (10). Brain natriuretic peptide (BNP) is produced by the ventricles in response to an increase in myocardial stretch, damage, or ischemia (10). N-terminal pro-brain natriuretic peptide (NT-proBNP) is the biologically inactive peptide that is cleaved off the pro-hormone, proBNP (11). The diagnostic and prognostic power of both BNP and NT-proBNP is similar, however, NT-proBNP is less sensitive to breakdown than BNP is, which results in a more accurate measurement and reproducibility (11). Plasma levels of NT-proBNP have shown to relate to a low peak oxygen uptake (peak VO_2_) in HF patients (12).

Serum levels of CRP and NT-proBNP have shown to predict adverse outcomes in patients with HF, both in HF with reduced ejection fraction (HFrEF, LVEF <50%) and in HF with preserved ejection fraction (HFpEF, LVEF >50%). Although CRP and NT-proBNP provide independent and complementary insight into CRF, whether CRP and/or NT-proBNP also independently predict the degree of CRF impairment in HF patients is not yet established. We hypothesize that two biomarkers, CRP, and NT-proBNP, by acting as surrogates for different pathophysiologic mechanisms, inflammation and myocardial strain, respectively, will independently predict the degree of CRF impairment in patients with HF across the spectrum of LVEF including both HFrEF and HFpEF. The objective of this study was to investigate whether CRP and/or NT-proBNP can independently predict CRF impairment, defined as reduced peak VO_2_ in patients with HF across a wide range of ejection fraction.

## Materials and Methods

### Study Design

We retrospectively queried a database of de-identified data that was prospectively collected data from patients with symptomatic HF who completed one or more cardiopulmonary exercise tests (CPX) using a symptom-limited ramp protocol on a treadmill and had paired measurements of serum high-sensitivity CRP and NT-proBNP on the same day. All members of the research team have completed training on the ethical conduct of research on human subjects. The VCU Institutional Review Board approved of the study, which was conducted according to the International Conference on Harmonization Good Clinical Practice Guidelines and the Declaration of Helsinki.

### Cardiopulmonary Exercise Testing

All patients underwent maximal CPX with a certified exercise physiologist under the supervision of a physician with a metabolic cart connected to a treadmill (Vmax Encore, Viasys, Yora Linda, CA) using a ramp protocol, as described before (13). Oxygen and carbon dioxide sensors were calibrated before the test with known oxygen, nitrogen, and carbon dioxide concentrations and the flow sensor was calibrated using a 3-L syringe. Subjects were asked to exercise to maximal fatigue. Twelve-lead ECG measurements were done at baseline, throughout the test, and during recovery. Every 2 min blood pressure was measured using an automated exercise compatible device (Tango, SunTech Medical). Expired gases were sampled during exercise with a mouthpiece-mounted sensor and were analyzed continuously to measure oxygen consumption (VO_2_), carbon dioxide production (VCO_2_), and minute ventilation (VE). The peak VO_2_ (mlO_2_•kg^−1^•min^−1^) during exercise was defined as the highest 10-s average value for VO_2_ during the last 30 s of the exercise. Peak VO_2_ measured during exercise is the most objective variable for assessment of functional capacity as is an important prognostic indicator (3). American Heart Association/American College of Cardiology guidelines for exercise testing contraindications and termination criteria were followed.

### Doppler Echocardiography

Subjects underwent transthoracic Doppler echocardiogram. Echocardiography was performed according to the American Society of Echocardiography measurement guidelines and provides information on both cardiac dimensions and function (14). LV end-diastolic and end-systolic volumes (LVEDV, LVESV), EF, and early transmitral E wave velocities were obtained. Early mitral annulus (e′) velocities obtained by tissue Doppler were averaged between lateral and septal e′ and tricuspid annulus plane systolic excursion. The E/e′ ratio provides information on diastolic function and was calculated to estimate LV filling pressures (15, 16).

### Biomarker Analysis

We analyzed differential comprehensive metabolic profile and plasma levels of biomarkers including high-sensitivity CRP and NT-proBNP. CRP, a marker for systemic inflammation, is increased in HF and has shown to relate to poor exercise performance (8, 9). CRP values <3.0 mg/L were considered within normal range. CRP values >3.0 mg/L have shown to be associated with an increased risk of cardiovascular disease (6). N-terminal pro-brain natriuretic peptide (NT-proBNP), a biomarker of myocardial strain, correlates with exercise capacity in HF (12). NT-proBNP values <300 ng/ml were considered within normal range (17). Both CRP and NT-proBNP independently predict adverse outcomes in patients with HF (7, 18, 19). Furthermore, we analyzed White Blood Cell (WBC) count, absolute neutrophils, and leukocyte count. Neutrophil to leukocyte ratio (NLR) is a measure for systemic inflammation (20). A NLR >4 has prognostic value in cardiovascular disease (21).

### Data Analysis

For the data analysis, each of the tests were considered as a separate measurement for data analysis. We assessed the correlation between different clinical parameters and peak oxygen consumption (peak VO_2_), as the preferred measures of CRF obtained during effort-limited maximal cardiopulmonary exercise testing (CPX). This was done for the entire HF group and then analyzed separately for 2 groups of patients stratified according to LVEF <50% (HFrEF) or ≥50% (HFpEF). According to the Fick principle, peak VO_2_ is determined by stroke volume, heart rate, and arterial-venous oxygen difference and therefore reflects both cardiac, vascular, and peripheral skeletal muscle components. The cause of exercise intolerance has been proposed to be different in HFrEF and HFpEF patients, therefore we stratified according to LVEF (22).

### Statistical Analysis

Data was tested for deviation from Gaussian distribution using the Kolmogorov–Smirnov test, and due to a lack of deviation, is presented as mean and standard deviation. Data that deviated from Gaussian distribution is presented as median and interquartile range. For normally distributed continuous variables, differences between groups were evaluated using independent-samples *t*-test, and for not normally distributed variables with the Mann–Whitney *U*-test. Correlations between continuous variables were assessed using linear regression. Multivariate analysis using stepwise linear regression was used to assess predictors of CRF with available clinical parameters. A ROC curve analysis was performed to evaluate whether the biomarkers had discriminative value for reduced CRF defined as peak VO_2_ <10 mlO_2_•kg^−1^•min^−1^and peak VO_2_ <14 mlO_2_•kg^−1^•min^−1^. SPSS Statistics 24.0 (IBM, Armonk, NY) statistical software package was used for all analyses. No missing data imputation method used. A *p*-value < 0.05 was considered statistically significant.

## Results

**Figure 1:**
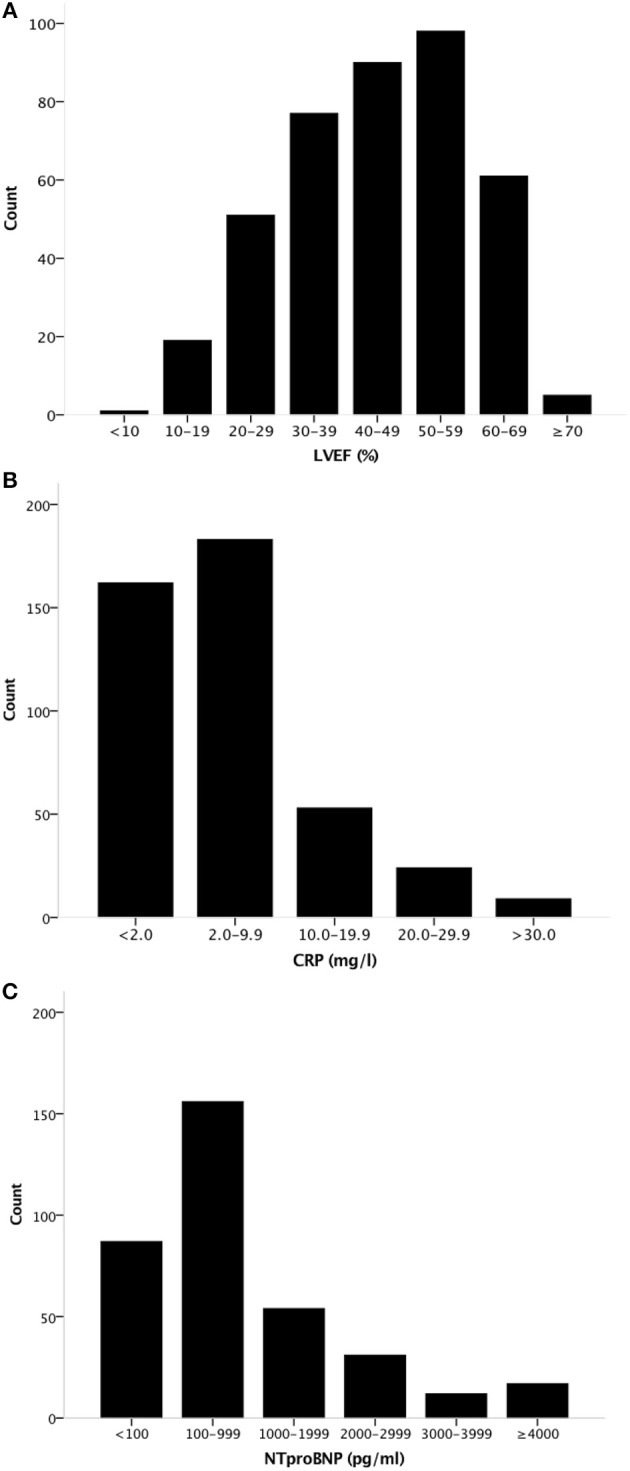
Distribution of LVEF, CRP, and NT-proBNP levels in the population. Histogram showing the distribution of **(A)** LVEF, **(B)** CRP levels, and **(C)** NT-proBNP levels in the population. LVEF, Left Ventricular Ejection Fraction.

We evaluated a total of 366 CPX from 200 different patients (2.0 ± 1.3 studies per patient) of which the clinical characteristics are shown in Table 1 for all patients. Clinical characteristics are shown separately for patients with HF with reduced LVEF (LVEF <50%) and for patients with HF with preserved LVEF HFpEF (LVEF ≥50%) in Table 2. Mean age of patients was 57 ± 10 years and 110 (55%) were male. Mean LVEF was 44 ± 14% (HFrEF, *N* = 109 and HFpEF, *N* = 57) (Figure 1), with a mean LVEF of 36 ± 11% in HFrEF, and 58 ± 6% in HFpEF. Median high-sensitivity CRP levels were 3.7 [1.5–9.0] mg/L, respectively and median NT-proBNP levels were 377 [106–1,464] pg/ml, respectively. In HFrEF, median CRP levels were 4.2 [1.9–9.2] mg/L, and median NT-proBNP levels were 1,029 [280–2,263] pg/ml. In HFpEF, median CRP levels were 3.7 [0.6–10.8] mg/L and median NT-proBNP levels were 102 [46–183] pg/ml, respectively (*P* < 0.001 for NT-proBNP, *P* = 0.03 for CRP between HFpEF and HFrEF). The distribution of LVEF, CRP, and NT-proBNP is shown in Figure 1. Mean White Blood Cell (WBC) count and median Neutrophil to Leukocyte Ratio (NLR) was 7.2 (2.4) × 10^9^/L and 2.3 [1.6–3.1], with a mean WBC count and median NLR of 7.4 (2.6) × 10^9^/L and 2.0 [1.5–2.7] in HFpEF, and 6.9 (2.1) × 10^9^/L and 2.3 [1.6–3.2] in HFrEF, respectively, (*P* = 0.198 for WBC count, *P* = 0.07 for NLR). Mean peak oxygen consumption (peak VO_2_) was 16 ± 4 mlO_2_•kg^−1^•min^−1^and mean treadmill exercise time (TET) was 8.7 ± 2.8 min.

**Table 1:** Clinical characteristics in all patients.

**Patient characteristics (*N* = 200)**	
Age, y	57 (10)
Male sex, *n* (%)	110 (55%)
BMI, kg/m^2^	35 (8)
**BIOMARKERS**	
CRP, mg/L	7.1 (8.9) 3.7 [1.5–9.0]
WBC, × 10^9^/L	7.2 (2.4)
Absolute Neutrophils, × 10^9^/L	4.0 [3.1–5.2]
Absolute Lymphocytes, × 10^9^/L	1.9 (0.6)
Neutrophil-to-Lymphocyte ratio	2.3 [1.6–3.1]
NT-proBNP, ng/ml	1306 (3092) 377 [106–1464]
**DOPPLER ECHOCARDIOGRAPHY PARAMETERS**	
LVEF, %	44 (14)
E/e'	15 (8)
**CARDIOPULMONARY EXERCISE PARAMETERS**	
Treadmill exercise time, min	8.7 (2.8)
Peak VO_2_, mlO_2_•kg^−1^•min^−1^	16 (4)
Peak VO_2_ % of predicted	54 (16)
VE/VCO_2_ slope	33 (7)
DASI score	30 (16)
MLWHF score	46 (26)

*BMI, Body Mass Index; WBC, White Blood Cell Count; NLR, Neutrophil to Leukocyte Ratio; DASI, Duke Activity Status Index; MLWHF, Minnesota Living With Heart Failure; LVEF, Left Ventricular Ejection Fraction; VO_2_, Oxygen uptake; VE, Minute Ventilation; VCO_2_, Carbon Dioxide output. Data shown as mean and (standard deviation) or median and [interquartile range]*.

**Table 2:** Clinical characteristics in HFpEF and HFrEF patients.

	**HFpEF (*N* = 57)**	**HFrEF (*N* = 109)**	
Age, y	53 (9)	57 (10)	*P* = 0.03
Male sex, n (%)	19 (33%)	78 (72%)	*P* < 0.001
BMI, kg/m^2^	40 (8)	34 (8)	*P* < 0.001
**BIOMARKERS**			
CRP, mg/L	6.4 (7.1) 3.7 [0.6–10.8]	7.7 (9.7) 4.2 [1.9–9.2]	*P* = 0.03
NT-proBNP, ng/ml	172 (266) 102 [46-183]	1906 (3693) 1029 [280–2263]	*P* < 0.001
HgB, g/dl	13.1 (1.8)	13.2 (1.7)	*P* = 0.75
HBA1c, %	7.7 (1.9)	6.8 (1.4)	*P* < 0.001
WBC, × 10^9^/L	7.4 (2.6)	6.9 (2.1)	*P* = 0.198
Absolute Neutrophils, × 10^9^/L	4.1 [3.0–5.4]	4.0 [3.1–5.2]	*P* = 0.71
Absolute Lymphocytes, × 10^9^/L	2.1 (0.6)	1.8 (0.6)	*P* = 0.002
Neutrophil-to-Lymphocyte ratio	2.0 [1.5–2.7]	2.3 [1.6–3.2]	*P* = 0.07
**DOPPLER ECHOCARDIOGRAPHY PARAMETERS**			
LVEF, %	58 (6)	36 (11)	*P* < 0.001
LVEDV, ml	110 (32)	172 (62)	*P* < 0.001
LVESV, ml	47 (19)	115 (55)	*P* < 0.001
E′	8.1 (2.4)	6.5 (3.6)	*P* = 0.01
E/e′	11.3 (4.7)	17.2 (8.3)	*P* < 0.001
**CARDIOPULMONARY EXERCISE PARAMETERS**			
Treadmill exercise time, min	9.4 (2.6)	8.4 (2.8)	*P* = 0.005
Peak VO_2_, mlO_2_•kg^−1^•min^−1^	16.5 (4.6)	15.4 (4.3)	*P* = 0.04
Peak VO_2_ % of predicted	55 (17)	52 (14)	*P* = 0.356
VE/VCO_2_ slope	30 (5)	33 (7)	*P* < 0.001

*BMI, Body Mass Index; HgB, Hemoglobin; HBA1c, Hemoglobin A1c; WBC, White Blood Cell Count; NLR, Neutrophil to Leukocyte Ratio; LVEF, Left Ventricular Ejection Fraction; LVEDV, Left Ventricular End Diastolic Volume; LVESV, Left Ventricular End Systolic Volume; VO_2_, Oxygen uptake; VE, Minute Ventilation; VCO_2_, Carbon Dioxide output. Data shown as mean and (standard deviation) or median and [interquartile range]*.

### CRP as a Predictor of CRF

**Figure 2:**
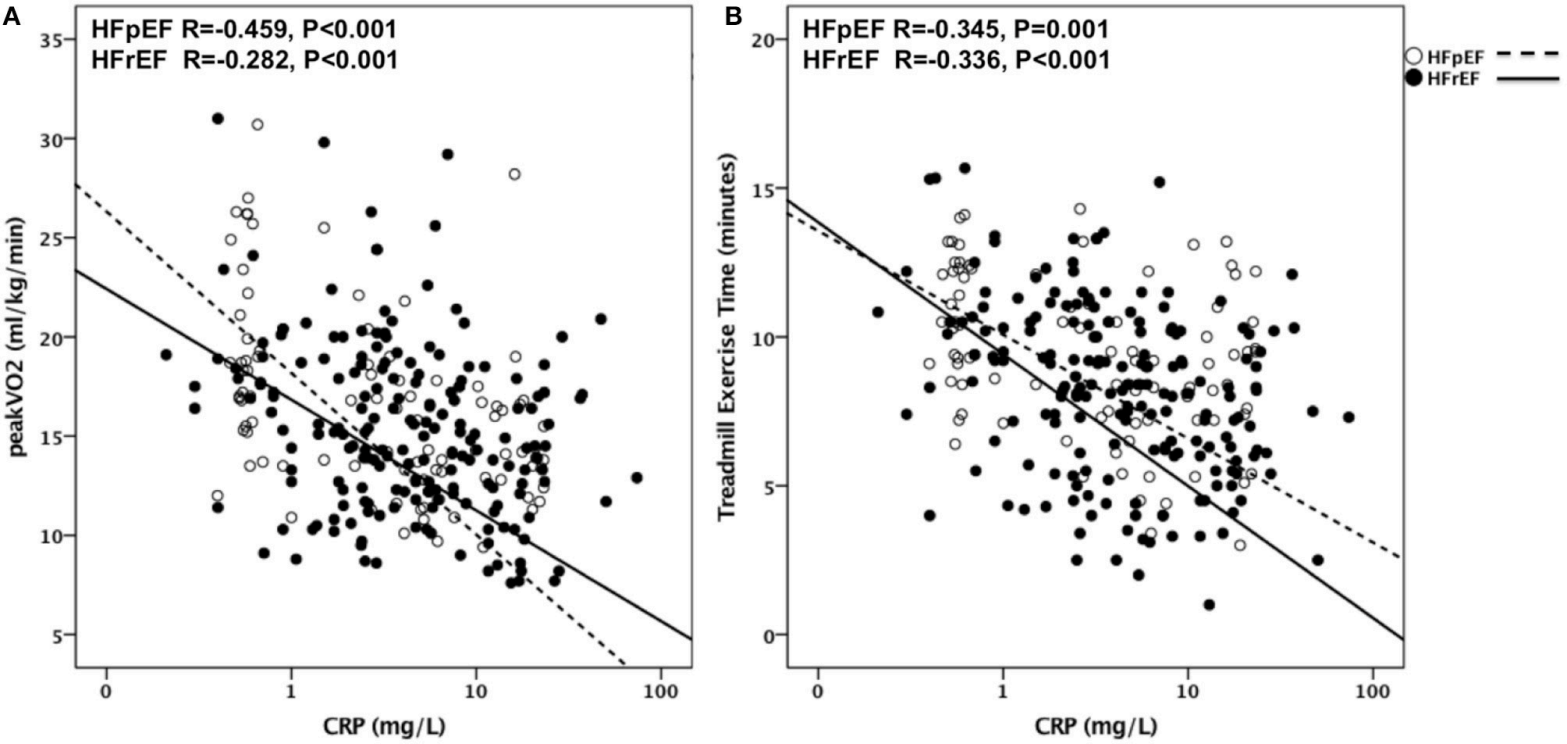
CRP as predictor of CRF in HFrEF and HFpEF. Correlations are shown in **(A)** between CRP and Peak VO_2_ in patients with HFpEF (*R* = −0.459, *P* < 0.001) and with HFrEF (*R* = −0.282, *P* < 0.001) and in **(B)** between CRP and TET in patients with HFpEF (*R* = −0.345, *P* < 0.001) and with HFrEF (*R* = −0.336, *P* < 0.001). VO_2_, Oxygen uptake; HFpEF, Heart Failure with preserved Ejection Fraction; HFrEF, Heart Failure with reduced Ejection Fraction; TET, Treadmill Exercise Time.

CRP levels significantly and inversely correlated with peak VO_2_ (*R* = −0.350, *p* < 0.001, *N* = 316), and with TET (*R* = −0.342, *p* < 0.001, *N* = 314) as shown in Figure 2. The association between CRP and peak VO_2_ and between CRP and TET remained significant when the analysis was limited to HFrEF patients (*R* = −0.282, *p* < 0.001, *N* = 192 for peak VO_2_ and *R* = 0.336, *p* < 0.001, *N* = 190 for TET) and to HFpEF patients (*R* = −0.459, *p* < 0.001, *N* = 90 for peak VO_2_ and *R* = −0.345, *p* < 0.01, *N* = 90 for TET).

### NT-proBNP as a Predictor of CRF

**Figure 3:**
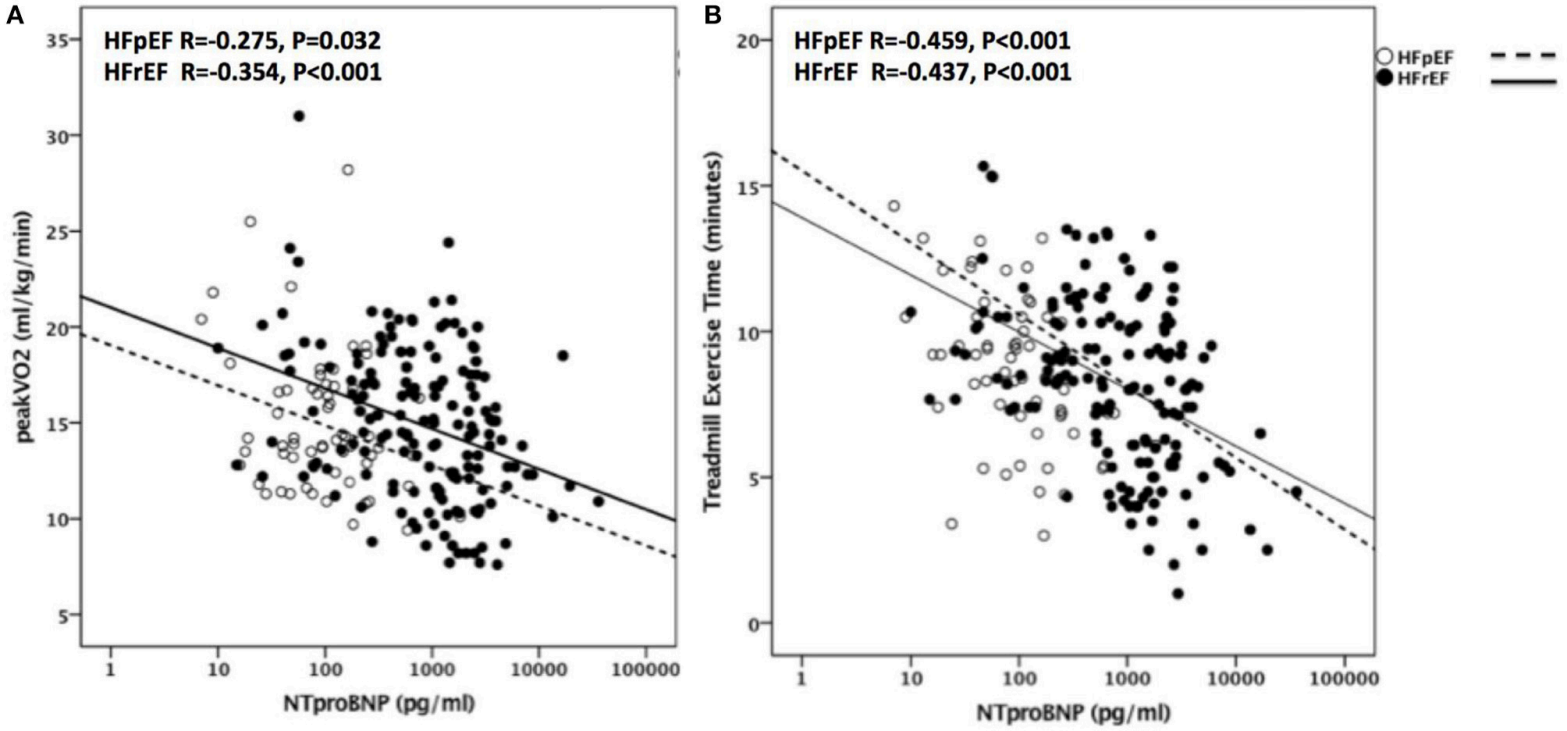
NT-proBNP as predictor of CRF in HFrEF and HFpEF. Correlations are shown in **(A)** between NT-proBNP and Peak VO_2_ in patients with HFpEF (*R* = −0.275, *P* = 0.032) and with HFrEF (*R* = −0.354, *P* < 0.001) and in **(B)** between NT-proBNP and TET in patients with HFpEF (*R* = −0.459, *P* < 0.001) and with HFrEF (*R* = −0.437, *P* < 0.001). VO_2_, oxygen uptake; HFpEF, Heart Failure with preserved Ejection Fraction; HFrEF, Heart Failure with reduced Ejection Fraction; TET, Treadmill Exercise Time.

NT-proBNP levels significantly and inversely correlated with peak VO_2_ (*R* = −0.330, *p* < 0.001, *N* = 258) and with TET (*R* = −0.412, *p* < 0.001, *N* = 256) as shown in Figure 3. The association between NT-proBNP and peak VO_2_ and between NT-proBNP and TET remained significant when the analysis was limited to HFrEF patients (*R* = −0.354, *p* < 0.001, *N* = 168 for peak VO_2_ and *R* = −0.437, *p* < 0.001, *N* = 166 for TET) as well as in HFpEF patients (*R* = −0.275, *p* = 0.032, *N* = 61 for peak VO_2_ and *R* = −0.459, *p* < 0.001, *N* = 61 for TET).

### Multivariate Analysis

Multivariate analysis including CRP and NT-proBNP showed that both CRP and NT-proBNP independently predicted peak VO_2_ (*R* = 0.421, *p* < 0.001 and *p* < 0.001, respectively), and TET (*R* = 0.478, *p* < 0.001, and *p* < 0.001, respectively). CRP and NT-proBNP did not exhibit collinearity (*R* = +0.05, *p* = 0.426, *N* = 255). When the analysis was limited to HFrEF patients, CRP and NT-proBNP predicted peak VO_2_ (*R* = 0.432, *p* < 0.001, *p* < 0.001, respectively) and TET (*R* = 0.496, *p* < 0.001, *p* < 0.001, respectively). When the analysis was limited to HFpEF patients, NT-proBNP predicted peak VO_2_ independent from CRP (*R* = 0.287, *p* = 0.5 for CRP, *p* = 0.04 for NT-proBNP) and TET (*R* = 0.459, *p* = 0.988 for CRP, *p* < 0.001 for NT-proBNP). Furthermore, multivariate analysis including NT-proBNP, BMI, age, and sex significantly predicted peak VO_2_ (*R* = 0.631, *p* < 0.001, *p* < 0.001, *p* = 0.04, respectively, with a trend for sex (*p* = 0.08). NT-proBNP, CRP, BMI, age, and sex significantly predicted TET (*R* = 0.651, *p* < 0.001, *p* = 0.006, *p* < 0.001, *p* < 0.001, *p* = 0.029, respectively). When the analysis was limited to HFrEF patients, BMI, age, sex, NT-proBNP, and CRP predicted Peak VO_2_ (*R* = 0.669, *p* < 0.001, *p* < 0.001, *p* < 0.001, *p* < 0.001, *p* = 0.03, respectively) and TET (*R* = 0.716, *p* < 0.001, *p* < 0.001, *p* < 0.001, *p* < 0.001, *p* = 0.002, respectively). When the analysis was limited to HFpEF patients, BMI, and age significantly predicted peak VO_2_ (*R* = 0.669, *p* < 0.001, and *p* < 0.001, respectively) and BMI, age, and NT-proBNP predicted TET (*R* = 0.680 *p* < 0.001, *p* = 0.017, *p* = 0.03, respectively).

### ROC Curve Analysis

**Figure 4:**
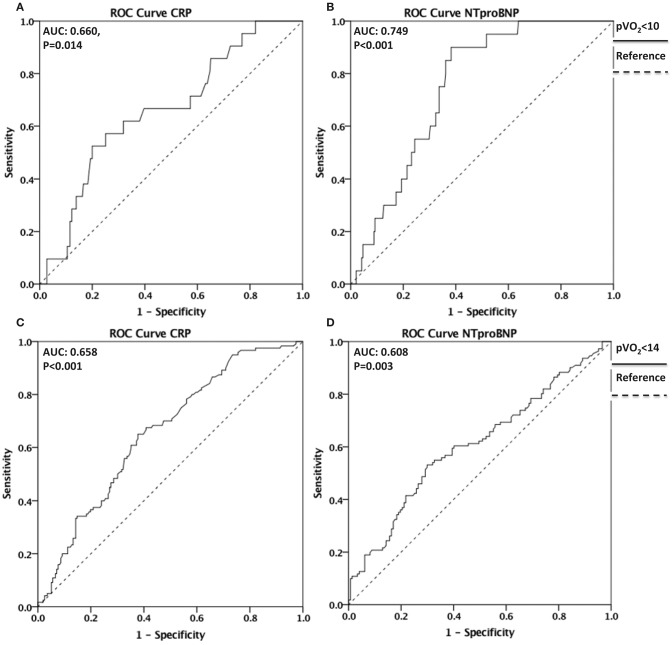
Discriminative value of CRP and NT-proBNP for reduced CRF. ROC curves depicting the discriminative value of CRP in **(A)** and of NT-proBNP in **(B)** for reduced CRF defined as Peak VO_2_ <10 mlO_2_•kg^−1^•min^−1^(AUC = 0.660, *P* = 0.014 for CRP and AUC = 0.749, *P* < 0.001 for NT-proBNP). ROC curves depicting the discriminative value of CRP in **(C)** and NT-proBNP in **(D)** for reduced CRF defined as Peak VO_2_ <14 mlO_2_•kg^−1^•min^−1^ (AUC = 0.658, *P* < 0.001 for CRP and AUC = 0.608, *P* = 0.003 for NT-proBNP). ROC, Receiver operating characteristic; CRF, Cardiorespiratory Fitness; VO_2_, oxygen uptake; AUC. Area Under the Curve.

A ROC curve analysis was performed to evaluate whether CRP and NT-proBNP have discriminative value for reduced CRF, defined as peak VO_2_ <10 mlO_2_•kg^−1^•min^−1^and peak VO_2_ <14 mlO_2_•kg^−1^•min^−1^(Figure 4). For peak VO_2_ <10 mlO_2_•kg^−1^•min^−1^, the area under the curve (AUC) = 0.660 95% CI [0.544–0.776], *P* = 0.014 for CRP and AUC = 0.749 95% CI [0.669–0.829], *P* < 0.001 for NT-proBNP. For peak VO_2_ <14 mlO_2_•kg^−1^•min^−1^, AUC = 0.658 95% CI [0.597–0.718], *P* < 0.001 for CRP and AUC = 0.608 95% CI [0.537–0.678] *P* = 0.003 for NT-proBNP.

## Discussion

CRF is an important determinant of quality of life and prognostic indicator in patients with HF (23, 24). In the current study, we show that systemic inflammation, as measured by elevated CRP levels, and myocardial strain, as indicated by elevated NT-proBNP levels, independently predict impaired CRF in patients with HF, reflected in reduced peak VO_2_. Both CRP and NT-proBNP show a high discriminative value for reduced CRF as defined by peak VO_2_ <10 mlO_2_•kg^−1^•min^−1^and peak VO_2_ <14 mlO_2_•kg^−1^•min^−1^.

### Systemic Inflammation in Heart Failure

HF is characterized by systemic inflammation, as shown by elevated circulating levels of inflammatory biomarkers in patients that increase with progression of the disease (25). For one, systemic inflammation may be the result of HF by means of tissue hypoperfusion and neurohormonal activation (26), or inflammation may play a pathophysiologic role in HF (27, 28). It is suggested that the pro-inflammatory state contributes to the development and progression of HF, not only by impairing myocardial function but also by affecting other organs and tissues and thereby adding to other aspects of the HF syndrome including cachexia and anemia (25).

The preferred inflammatory biomarker in cardiovascular disease is CRP (29, 30). HF patients have elevated CRP levels and such levels tend to increase with clinical decompensation and predict worse outcomes (5, 31, 32). Elevated CRP levels reflect inflammatory and immune deregulation in HF (31, 33). Elevated CRP levels also correlate with worse cardiac function (34, 35), and worse functional capacity in patients with ischemic heart disease and systolic HF (8, 9). The association between elevated CRP levels and HF is however complex and likely only incompletely understood. CRP is produced in the liver in response to cytokines such as Interleukin-6 (IL-6) (36). IL-6 is considered a secondary cytokine produced by myeloid cells in response to Interleukin-1 (IL-1) and Tumor Necrosis Factor-α (TNF-α) (36). Elevated IL-1 and TNF-α levels have been reported in patients with HF (33, 37). Moreover, IL-1 and TNF-α are also known as soluble cardiodepressant factors in patients with sepsis (38). More recently enhanced IL-1 activity has been described in patients with acute decompensated HF (31). IL-1 induces a reversible left ventricular systolic dysfunction in the mouse that is characterized by β-adrenergic receptor desensitization and impaired contractile reserve (39). This data points to an active role of IL-1 in the pathophysiology of HF.

### Myocardial Strain

Elevated NT-proBNP levels reflect myocardial strain due to increased pressure, however, levels may also increase in response to other insults such as ischemia or inflammatory cytokines. NT-proBNP has shown to predict adverse outcomes in HF patients (10). Although BNP exerts protective effects on the heart during HF, the circulating levels of BNP or NT-proBNP reflect worse hemodynamics (elevated filling pressures) and neurohormonal activation (40). A lack of endogenous BNP response facilitates the onset of HF in animal experimental models (41), whereas potentiation of the BNP effects using recombinant BNP or neprylisin inhibitors help prevent HF (42).

### Limitations

The retrospective nature of the study limited the power of our analyses, specifically in the HFpEF cohort for which the sample size was smaller than for HFrEF patients. Another limitation of our study is that every visit in the data analysis was used as separate data point, which results in some patients being represented more than once in the database. Having patients represented more than once can alter the representation of the patient population, as a single patient could have provided more than one data entry leading to an over-representation of their specific clinical characteristics. Lastly, we were not able to address the role of other biomarkers for inflammation in HF in our study.

### Potential Implications for Diagnosis, Risk Stratification, and Treatment

According to our findings, both elevated CRP and NT-proBNP levels are independently predictive of impaired CRF in HF and predictive of moderately or severe reduction in peak VO_2_. A scoring system that would include both biomarkers is therefore likely to yield a better discrimination than only one of the markers. In non-ST elevation ACS, CRP, and BNP in combination with troponin I were predictive of mortality, MI and CHF. Further, a combination of the biomarkers provided additional prognostic value (43). These observations were validated in a cohort of 1,635 patients in the TACTICS-TIMI 18 study, and after adjustment for known clinical predictors, the number of elevated biomarkers remained predictive of the composite end point. Specifically, patients with one, two, and three elevated biomarkers had a 2.1, 3.1, and 3.7-fold increase in the risk of death, MI, or development of CHF after 6 months (43). A scoring system that could utilize the prognostic power of both CRP and NT-proBNP would allow for risk stratification beyond that solely provided by each of the markers used separately to predict CRF in patients with HF across a wide range of LVEF.

Inhibiting systemic inflammation with anti-inflammatory therapies and alleviating myocardial strain may represent two independent therapeutic strategies to improve CRF in patients with HF. Phase II studies have started exploring the effects of Interleukin-1 blockers in HF. A pilot feasibility study in 7 patients was conducted to test the efficacy of Anakinra on CP exercise performance in patients with HF and evidence of CRP. CRP levels were greatly reduced and peak VO_2_ significantly improved (31). In the REDHART sub study, 60 patients with HFrEF and elevated CRP were randomly assigned to daily subcutaneous injections of Anakinra 100 mg for weeks, 12 weeks, or placebo (44). Treatment with Anakinra did not affect peak VO_2_ or VE/VCO_2_ slope at 2 weeks; however, patients showed improvement in peak VO_2_ when assigned to the 12-week group. Further, the incidence of death or rehospitalization for HF at 24 weeks was 6, 31, and 30% for the Anakinra 12-week, Anakinra 2-week, and placebo groups, respectively. In the D-HART pilot study, the effects of IL-1 blockade with Anakinra on aerobic exercise capacity and CRP in patients with HFpEF were examined (45). Anakinra led to a statistically significant improvement in peak VO_2_ consumption and a significant reduction in plasma CRP levels.

In a follow up study, the Diastolic Heart Failure Anakinra Response Trial-2 (DHART-2), patients with stable symptomatic HFpEF were treated with Anakinra to confirm the effects on peak VO_2_ and CRP and observe its effects on serum NT-proBNP (46). Twenty-eight patients completed two visits or more and Anakinra was found to significantly reduce CRP as well as NT-proBNP levels. After 12 weeks of IL-1 blockade with Anakinra, NT-proBNP was reduced at a magnitude that correlated with CRP reduction. Anakinra however failed to increase peak VO_2_ in the DHART2 study. The potential benefits of IL-1 blockade in patients with heart disease is further supported by the results of the phase III Canakinumab Antiinflammatory Thrombosis Outcome Study (CANTOS) (47), in which patients with prior acute myocardial infarction were randomized to canakinumab, IL-1β blocker, or placebo, and showed a significant reduction in major adverse cardiac events. A small single-center sub-study of the CANTOS trial showed a significant improvement in peak VO_2_ in canakinumab-treated patients at 3 and 12 months (46).

Neprilysin inhibitors have also provided a novel therapeutic strategy to combat HF symptoms and promote CRF. Another study was completed to compare the effects of Candoxatril (novel neutral endopeptidase inhibitor) with those of Furosemide in the treatment of patients with mild HF (37). Male patients with mild HF were randomly assigned to treatment with 20 mg of Furosemide twice a day, 200 mg of Candoxatril twice a day, or 400 mg of Candoxatril twice a day, for 9 days. For patients assigned to Furosemide, treadmill exercise capacity decreased by 30 ± 26 s compared to an increase of 12 ± 35 and of 35 ± 31 s for 200 mg of Candoxatril twice a day and 400 mg of Candoxatril twice a day, respectively (37). A pilot study was completed to evaluate the short-term effects of sacubitril/valsartan on maximal exercise capacity evaluated by peak VO_2_ in stable patients with symptomatic HFrEF, with a secondary end point looking at changes in the VE/VCO_2_ slope. When compared with baseline peak VO_2_, patients experienced a significant increase in peak VO_2_ at 30 days (+0.92 mlO_2_•kg^−1^•min^−1^), which corresponded to a 7.9% increase (38). These beneficial effects on CRF, fit well with the overall favorable effects of sacubitril/valsartan on major adverse cardiovascular events and cardiac death in the Prospective Comparison of ARNI (Angiotensin Receptor-Neprilysin Inhibitor) with ACEI (Angiotensin-Converting-Enzyme Inhibitor) to Determine Impact on Global Mortality and Morbidity in Heart Failure (PARADIGM-HF) trial (48).

## Conclusion

Biomarkers of inflammation and myocardial strain independently predict reduced peak VO_2_ in HF patients. Anti-inflammatory therapies and therapies alleviating myocardial strain may independently improve CRF in HF patients across a large spectrum of LVEF.

## Ethics Statement

The study was completed with a de-identified database available at VCU. The research was considered exempt from Institutional Review Board review as per VCU IRB guidelines.

## Author Contributions

JC, SC, CT, DK, LB, HB, GW, BV, and AA clinical data collection. JW, KR, and AA database analysis, drafting of the manuscript. LB, MB, MV, and RA critical revision of the manuscript.

### Conflict of Interest Statement

AA has served as a consultant to Novartis, Swedish Orphan Biovitrum, Janssen, Merck, and Olatec. BV has served as consultant to Novartis. The remaining authors declare that the research was conducted in the absence of any commercial or financial relationships that could be construed as a potential conflict of interest.

## Abbreviations

CRF: Cardiorespiratory Fitness
HF: Heart Failure
HFrEF: Heart Failure with Reduced Ejection Fraction
HFpEF: Heart Failure with Preserved Ejection Fraction
METs: Metabolic Equivalents
VO_2_: Oxygen Consumption
CRP: C-Reactive Protein
NT-proBNP: N-Terminal Pro-Brain Natriuretic Peptide
WBC: White Blood Cell
NLR: Neutrophil to Leukocyte Ratio
ANP: Atrial Natriuretic Peptide
CVD: Cardiovascular Disease
CVP: Central Venous Pressure
RAAS: Renin Angiotensin Aldosterone System
LVEF: Left Ventricular Ejection Fraction
LVEDV: Left Ventricular End Diastolic Volume
LVESV: Left Ventricular End Systolic Volume
CPX: Cardiopulmonary Exercise Test
TET: Treadmill Exercise Time
Peak VO_2_: Peak Oxygen Consumption
VE/VCO_2_: Ventilatory Efficiency
ACS: Acute Coronary Syndrome
MI: Myocardial Infarction
ARNI: Angiotensin Receptor-Neprilysin Inhibitor
ACEI: Angiotensin-Converting-Enzyme Inhibitor
PARADIGM-HF: Prospective Comparison of ARNI with ACEI to Determine Impact on Global Mortality and Morbidity in Heart Failure
CANTOS: Canakinumab Antiinflammatory Thrombosis Outcome Study
ROC: Receiver Operating Characteristic
AUC: Area Under the Curve.

## References

[1] Kondamudi N Haykowsky M Forman DE Berry JD Pandey A. Exercise training for prevention and treatment of heart failure. Prog Cardiovasc Dis. (2017) 60:115–20. 10.1016/j.pcad.2017.07.001 28684221

[2] Lavie CJ Arena R Swift DL Johannsen NM Sui X Lee DC. Exercise and the cardiovascular system: clinical science and cardiovascular outcomes. Circ Res. (2015) 117:207–19. 10.1161/CIRCRESAHA.117.305205 26139859 PMC4493772

[3] Balady GJ Arena R Sietsema K Myers J Coke L Fletcher GF. Clinician's guide to cardiopulmonary exercise testing in adults: a scientific statement from the American heart association. Circulation (2010) 122:191–225. 10.1161/CIR.0b013e3181e52e69 20585013

[4] Libby P Ridker PM Maseri A. Inflammation and atherosclerosis. Circulation (2002) 105:1135–43. 10.1161/hc0902.104353 11877368

[5] Van Tassell BW Abouzaki NA Oddi Erdle C Carbone S Trankle CR Melchior RD. Interleukin-1 blockade in acute decompensated heart failure: a randomized, double-blinded, placebo-controlled pilot study. J Cardiovasc Pharmacol. (2016) 67:544–51. 10.1097/FJC.0000000000000378 26906034 PMC5749643

[6] Emerging Risk Factors Collaboration Kaptoge S Di Angelantonio E Lowe G Pepys MB Thompson SG. C-reactive protein concentration and risk of coronary heart disease, stroke, and mortality: an individual participant meta-analysis. Lancet (2010) 375:132–40. 10.1016/S0140-6736(09)61717-7 20031199 PMC3162187

[7] Ridker PM MacFadyen JG Everett BM Libby P Thuren T Glynn RJ. Relationship of C-reactive protein reduction to cardiovascular event reduction following treatment with canakinumab: a secondary analysis from the CANTOS randomised controlled trial. Lancet (2017) 391:319–28. 10.1016/S0140-6736(17)32814-3 29146124

[8] Canada JM Fronk DT Cei LF Carbone S Erdle CO Abouzaki NA. Usefulness of C-reactive protein plasma levels to predict exercise intolerance in patients with chronic systolic heart failure. Am J Cardiol. (2016) 117:116–20. 10.1016/j.amjcard.2015.10.020 26546248

[9] Rahimi K Secknus MA Adam M Hayerizadeh BF Fiedler M Thiery J. Correlation of exercise capacity with high-sensitive C-reactive protein in patients with stable coronary artery disease. Am Heart J. (2005) 150:1282–9. 10.1016/j.ahj.2005.01.006 16338272

[10] de Lemos JA McGuire DK Drazner MH. B-type natriuretic peptide in cardiovascular disease. Lancet (2003) 362:316–22. 10.1016/S0140-6736(03)13976-1 12892964

[11] Pan Y Li D Ma J Shan L Wei M. NT-proBNP test with improved accuracy for the diagnosis of chronic heart failure. Medicine (2017) 96:e9181. 10.1097/MD.0000000000009181 29390456 PMC5758158

[12] Felker GM Whellan D Kraus WE Clare R Zannad F Donahue M. N-terminal pro-brain natriuretic peptide and exercise capacity in chronic heart failure: data from the heart failure and a controlled trial investigating outcomes of exercise training (HF-ACTION) study. Am Heart J. (2009) 158(4 Suppl.):S37–44. 10.1016/j.ahj.2009.07.011 19782787 PMC3748954

[13] Arena R Humphrey R Peberdy MA Madigan M. Predicting peak oxygen consumption during a conservative ramping protocol implications for the heart failure population. J Cardiopulm Rehabil. (2003) 23:183–9. 10.1097/00008483-200305000-00004 12782901

[14] Porter TR Shillcutt SK Adams MS Desjardins G Glas KE Olson JJ. Guidelines for the use of echocardiography as a monitor for therapeutic intervention in adults: a report from the american society of echocardiography. J Am Soc Echocardiogr. (2015) 28:40–56. 10.1016/j.echo.2014.09.009 25559474

[15] Barmeyer A Müllerleile K Mortensen K Meinertz T. Diastolic dysfunction in exercise and its role for exercise capacity. Heart Fail Rev. (2009) 14:125–34. 10.1007/s10741-008-9105-y 18758943

[16] Terzi S Sayar N Bilsel T Enc Y Yildirim A Ciloglu F. Tissue doppler imaging adds incremental value in predicting exercise capacity in patients with congestive heart failure. Heart Vessels (2007) 22:237–44. 10.1007/s00380-006-0961-x 17653517

[17] van Kimmenade RR Pinto YM Bayes-Genis A Lainchbury JG Richards AM Januzzi JL. Usefulness of intermediate amino-terminal pro-brain natriuretic peptide concentrations for diagnosis and prognosis of acute heart failure. Am J Cardiol. (2006) 98:386–90. 10.1016/j.amjcard.2006.02.043 16860029

[18] Yin WH Chen JW Jen HL Chiang MC Huang WP Feng AN. Independent prognostic value of elevated high-sensitivity C-reactive protein in chronic heart failure. Am Heart J. (2004) 147:931–8. 10.1016/j.ahj.2003.11.021 15131554

[19] Park JJ Choi DJ Yoon CH Oh IY Jeon ES Kim JJ. Prognostic value of C-reactive protein as an inflammatory and n-terminal probrain natriuretic peptide as a neurohumoral marker in acute heart failure (from the Korean heart failure registry). Am J Cardiol. (2014) 113:511–7. 10.1016/j.amjcard.2013.10.022 24315115

[20] Ackland GL Minto G Clark M Whittle J Stephens RCM Owen T. Autonomic regulation of systemic inflammation in humans: a multi-center, blinded observational cohort study. Brain Behav Immun. (2018) 67:47–53. 10.1016/j.bbi.2017.08.010 28807718

[21] Uthamalingam S Patvardhan EA Subramanian S Ahmed W Martin W Daley M. Utility of the neutrophil to lymphocyte ratio in predicting long-term outcomes in acute decompensated heart failure. Am J Cardiol. (2011) 107:433–8. 10.1016/j.amjcard.2010.09.039 21257011

[22] Dhakal BP Malhotra R Murphy RM Pappagianopoulos PP Baggish AL Weiner RB. Mechanisms of exercise intolerance in heart failure with preserved ejection fraction: the role of abnormal peripheral oxygen extraction. Circ Hear Fail. (2015) 8:286–94. 10.1161/CIRCHEARTFAILURE.114.001825 25344549 PMC5771713

[23] Kupsky DF Ahmed AM Sakr S Qureshi WT Brawner CA Blaha MJ. Cardiorespiratory fitness and incident heart failure: the henry ford exercise testing (FIT) project. Am Heart J. (2017) 185:35–42. 10.1016/j.ahj.2016.12.006 28267473

[24] Szlachcic J Massie BM Kramer BL Topic N Tubau J. Correlates and prognostic implication of exercise capacity in chronic congestive heart failure. Am J Cardiol. (1985) 55:1037–42. 10.1016/0002-9149(85)90742-8 3984864

[25] Yndestad A Damås JK Oie E Ueland T Gullestad L Aukrust P. Systemic inflammation in heart failure - the whys and wherefores. Heart Fail Rev. (2006) 11:83–92. 10.1007/s10741-006-9196-2 16819581

[26] Van Linthout S Tschöpe C Inflammation—cause or consequence of heart failure or both? Curr Heart Fail Rep. (2017) 14:251–65. 10.1007/s11897-017-0337-9 28667492 PMC5527060

[27] Buckley LF Abbate A. Interleukin-1 blockade in cardiovascular diseases: a clinical update. Eur Heart J. (2018) 39:2063–9. 10.1093/eurheartj/ehy128 29584915

[28] Buckley LF Abbate A. Interleukin-1 blockade in cardiovascular diseases: from bench to bedside. BioDrugs (2018) 32:111–8. 10.1007/s40259-018-0274-5 29549570

[29] Pearson TA Mensah GA Wayne R Anderson JL CannonIII RO Criqui M. Markers of inflammation and cardiovascular disease. Circulation (2003) 107:499–511. 10.1161/01.cir.0000052939.59093.45. 12551878

[30] Ridker PM. Clinical application of C-reactive protein for cardiovascular disease detection and prevention. Circulation (2003) 107:363–9. 10.1161/01.CIR.0000053730.47739.3C 12551853

[31] Van Tassell BW Arena RA Toldo S Mezzaroma E Azam T Seropian IM. Enhanced interleukin-1 activity contributes to exercise intolerance in patients with systolic heart failure. PLoS ONE (2012) 7:e33438. 10.1371/journal.pone.0033438 22438931 PMC3306393

[32] Elster SK Braunwald E Wood HF. A study of C-reactive protein in the serum of patients with congestive heart failure. Am Heart J. (1956) 51:533–41. 10.1016/0002-8703(56)90099-0 13302128

[33] Testa M Yeh M Lee P Fanelli R Loperfido F Berman JW Circulating levels of cytokines and their endogenous modulators in patients with mild to severe congestive heart failure due to coronary artery disease or hypertension. J Am Coll Cardiol. (1996) 28:964–71. 10.1016/S0735-1097(96)00268-9 8837575

[34] Shah SJ Marcus GM Gerber IL McKeown BH Vessey JC Jordan MV. High-sensitivity C-reactive protein and parameters of left ventricular dysfunction. J Card Fail. (2006) 12:61–5. 10.1016/j.cardfail.2005.08.003 16500582

[35] Tang WH Shrestha K Van Lente F Troughton RW Martin MG Borowski AG. Usefulness of C-reactive protein and left ventricular diastolic performance for prognosis in patients with left ventricular systolic heart failure. Am J Cardiol. (2008) 101:370–3. 10.1016/j.amjcard.2007.08.038 18237602

[36] Ridker PM. C-Reactive protein: Eighty eighty years from discovery to emergence as a major risk marker for cardiovascular disease. Clin Chem. (2009) 55:209–15. 10.1373/clinchem.2008.119214 19095723

[37] Vasan RS Sullivan LM Roubenoff R Dinarello CA Harris T Benjamin EJ. Inflammatory markers and risk of heart failure in elderly subjects without prior myocardial infarction: the framingham heart study. Circulation (2003) 107:1486–91. 10.1161/01.CIR.0000057810.48709.F6 12654604

[38] Kumar A Thota V Dee L Olson J Uretz E Parrillo JE Tumor necrosis factor alpha and interleukin 1 beta are responsible for in vitro myocardial cell depression induced by human septic shock serum. J Exp Med. (1996) 183:949–58. 10.1084/jem.183.3.949 8642298 PMC2192364

[39] Van Tassell BW Seropian IM Toldo S Mezzaroma E Abbate A. Interleukin-1β induces a reversible cardiomyopathy in the mouse. Inflamm Res. (2013) 62:637–40. 10.1007/s00011-013-0625-0 23649041

[40] Díez J. Chronic heart failure as a state of reduced effectiveness of the natriuretic peptide system: implications for therapy. Eur J Heart Fail. (2017) 19:167–76. 10.1002/ejhf.656 27766748 PMC5297869

[41] Tamura N Ogawa Y Chusho H Nakamura K Nakao K Suda M. Cardiac fibrosis in mice lacking brain natriuretic peptide. Proc Natl Acad Sci U.S.A. (2000) 97:4239–44. 10.1073/pnas.070371497 10737768 PMC18212

[42] Gong X Mou Z Shao L Zou Y Gu Y Sun S. Human recombinant-B-type natriuretic peptide protect ventricular function and structure in ST-elevation myocardial infarction. Int J Clin Exp Pathol. (2015) 8:11622–8. 26617900 PMC4637716

[43] Sabatine MS Morrow DA de Lemos JA Gibson CM Murphy SA Rifai N. Multimarker approach to risk stratification in non-ST elevation acute coronary syndromes. Circulation (2002) 105:1760–3. 10.1161/01.CIR.0000015464.18023.0A 11956114

[44] Van Tassell BW Canada J Carbone S Trankle C Buckley L Oddi Erdle C. Interleukin-1 blockade in recently decompensated systolic heart failure: Results from REDHART (Recently Decompensated Heart Failure Anakinra Response Trial). Circ Hear Fail. (2017) 10:e004373. 10.1161/CIRCHEARTFAILURE.117.004373 29141858 PMC5699505

[45] Van Tassell BW Arena R Biondi-Zoccai G Canada JM Oddi C Abouzaki NA. Effects of interleukin-1 blockade with anakinra on aerobic exercise capacity in patients with heart failure and preserved ejection fraction (from the D-HART Pilot Study). Am J Cardiol. (2014) 113:321–7. 10.1016/j.amjcard.2013.08.047 24262762 PMC4899612

[46] Trankle C Canada J Carbone S Buckley LF Del Buono MG Christopher S Interleukin-1 blockade reduces Nt-probnp serum levels in patients with stable heart failure with preserved ejection fraction. J Am Coll Cardiol. (2018) 71:A869 10.1016/S0735-1097(18)31410-4

[47] Ridker PM Everett BM Thuren T MacFadyen JG Chang WH Ballantyne C. Antiinflammatory therapy with canakinumab for atherosclerotic disease. N Engl J Med. (2017) 377:1119–31. 10.1056/NEJMoa1707914 28845751

[48] McMurray JJ Packer M Desai AS Gong J Lefkowitz MP Rizkala AR. Angiotensin–neprilysin inhibition versus enalapril in heart failure. N Engl J Med. (2014) 371:993–1004. 10.1056/NEJMoa1409077 25176015

